# Molecular Analysis of the Enteric Protozoa Associated with Acute Diarrhea in Hospitalized Children

**DOI:** 10.3389/fcimb.2017.00343

**Published:** 2017-08-02

**Authors:** Sonia Boughattas, Jerzy M. Behnke, Khalid Al-Ansari, Aarti Sharma, Wafa Abu-Alainin, Asma Al-Thani, Marawan A. Abu-Madi

**Affiliations:** ^1^Department of Biomedical Science, College of Health Sciences, Biomedical Research Center, Qatar University Doha, Qatar; ^2^School of Life Sciences, University of Nottingham, University Park Nottingham, United Kingdom; ^3^Hamad Medical Corporation, Paediatric Emergency Center Doha, Qatar; ^4^Molecular Genetics Laboratory, Department of Laboratory Medicine and Pathology, Hamad Medical Corporation Doha, Qatar

**Keywords:** protozoa, diarrhea, pediatrics, Qatar, RT-PCR, statistical analysis, *Cryptosporidium*, genotyping

## Abstract

Pediatric diarrhea is a common cause of death among children under 5 years of age. In the current study, we investigated the frequency of intestinal parasites among 580 pediatric patients with chronic diarrhea. Parasitic protozoa (all species combined) were detected by molecular tools in 22.9% of the children and the most common parasite was *Cryptosporidium* spp. (15.1%). *Blastocystis hominis* was detected in 4.7%, *Dientamoeba fragilis* in 4%, *Giardia duodenalis* in 1.7%, and *Entamoeba histolytica* in 0.17%. Protozoan infections were observed among all regional groups, but prevalence was highest among Qatari subjects and during the winter season. Typing of *Cryptosporidium* spp. revealed a predominance of *Cryptosporidium parvum* in 92% of cases with mostly the IIdA20G1 subtype. Subtypes IIdA19G2, IIdA18G2, IIdA18G1, IIdA17G1, IIdA16G1, and IIdA14G1 were also detected. For *Cryptosporidium hominis*, IbA10G2 and IbA9G3 subtypes were identified. This study provides supplementary information for implementing prevention and control strategies to reduce the burden of these pediatric protozoan infections. Further analyses are required to better understand the local epidemiology and transmission of *Cryptosporidium* spp. in Qatar.

## Introduction

Diarrhoeal disease is the world's second leading cause of death among young children (UNICEF, 2013). The primary cause of diarrhea is the ingestion of infectious agents, bacterial, viral, or protozoan, from human or animal feces. Transmission occurs through anthroponotic, zoonotic, foodborne, and waterborne routes (Moore et al., 2016). It consists of ingestion of contaminated food or water, direct contact with infected feces, person-to-person contact, and poor personal hygiene (Boithias et al., 2016).

Intestinal protozoa that are most commonly associated with diarrhea in children include *Giardia duodenalis, Blastocystis hominis, Entamoeba histolytica, Cryptosporidium* spp., and *Dientamoeba fragilis* (Maas et al., 2014). Symptoms of infection follow an incubation period of approximately 1 week and include watery diarrhea, cramp, and nausea that may lead to inappetence and undernutrition, as observed in children in West Africa and South America (Quihui-Cota et al., 2015). The World Health Organization (WHO) has identified *Cryptosporidium* spp. as globally the most common diarrhea-causing protozoan (Pedersen et al., 2014), and the Center for Disease Control and Prevention has reported that giardiasis is experienced by approximately 33% of people in developing countries (CDC, 2015). Accordingly, due to their significant public health and socioeconomic implications, both parasites *Cryptosporidium* spp. and *G. duodenalis* were included in the WHO's “Neglected disease initiative” in 2004 (Osman et al., 2016). A wide diversity of *Cryptosporidium* spp. and subtypes infect humans and molecular analysis of the GP60 marker has been useful for identifying contamination sources and thus inferring their transmission routes (Cacciò and Chalmers, 2016). The cosmopolitan intestinal protozoa *B. hominis* and *D. fragilis* are both also of relevance in this context (Stark et al., 2016). *D. fragilis* is frequently detected as a co-infection with multiple protozoa (Garcia, 2016) especially with *B. hominis*, which is highly prevalent in Qatar (Abu-Madi et al., 2016a).

Conventional diagnosis of these protozoa relies widely on microscopical examination of stool samples. However, coproscopic identification is limited by a low sensitivity as evidenced by earlier studies reporting a marked difference in the prevalences of *B. hominis* and *G. duodenalis* when comparing the results of coproscopic and molecular analyses (Abu-Madi et al., 2015; Gotfred-Rasmussen et al., 2016). Moreover, coproscopic analysis is unable to distinguish between the pathogenic and non-pathogenic species of *Entamoeba* (Ali, 2015), between different species of *Cryptosporidium* and different assemblages of *Giardia* (Haque et al., 2009) as these are morphologically identical within genera. Hence there is an urgent need to adopt more sensitive and specific methods for species identification (Boughattas and Salehi, 2014), as accurate diagnosis will lead to early and more appropriate treatment (Maçin et al., 2016). By employing real-time PCR (RT-PCR), the aim of the current study was to investigate the frequency of intestinal parasites in pediatric patients with chronic diarrhea and to clarify the importance of individual species and relevant risk factors in such cases.

## Materials and methods

### Sample collection

A total of 580 stool samples were collected in sterile containers from diarrhoeal hospitalized pediatric patients during a whole year (March 2015–March 2016). Samples were transported to the Biomedical Research Center at Qatar University by ice box and stored at −20°C. The study was carried out in accordance with the recommendations of the Medical Research Centre and the Research Committee at Hamad Medical Corporation, Qatar (Research protocol # 16367/16). As the patients were young children, family members gave written informed consent in accordance with the Declaration of Helsinki. DNA was extracted using the QIAamp DNA stool minikit (Qiagen, Hilden, Germany) according to the manufacturer's instructions.

### RT-PCR detection

For the diagnosis of *Cryptosporidium* spp., *D. fragilis, G. duodenalis*, and *E. histolytica*, different sets of primers and probes were used as listed in Table 1. For *B. hominis*, SyberGreen chemistry was used for parasite identification (Abu-Madi et al., 2015).

**Table 1:** Primers and probes used in this study.

**Parasite**	**Target gene**	**Primers/Probes**	**Sequence 5′−3′**
Blastocystis hominis^*^	SSU rDNA	FwdS1	GGTCCGGTGAACACTTTGGATTT
		RvsS2	CCTACGGAAACCTTGTTACGACTTCA
*Giardia duodenalis*	SSU rRNA	Gd-80F	GACGGCTCAGGACAACGGTT
		Gd-127R	TTGCCAGCGGTGTCCG
		105T	FAM-CCCGCGGCG/ZEN/GTCCCTGCTAG
*Dientamoeba fragilis*	SSU rDNA	DF3	GTTGAATACGTCCCTGCCCTTT
		DF4	TGATCCAATGATTTCACCGAGTCA
		Probe	FAM-CACACCGCCCGTCGCTCCTA
*Entamoeba histolytica*	18S rRNA	Edh-239F	ATTGTCGTGGCATCCTAACTCA
		Edh-88R	GCGGACGGCTCATTATAACA
		96T	HEX-TCATTGAAT/ZEN/GAATTGGCCATTT
*Cryptosporidium* spp.	18S rRNA	SCL2	CAGTTATAGTTTACTTGATAATC
		SCR2	CAATACCCTACCGTCTAAAG
		CrySB	FAM/CCGTGGTAATTCTAGAGCTA/BHQ

* *No probe was used in the detection of this protozoan but SyberGreen chemistry*.

A unidirectional workflow pre- to post-PCR was enforced, and preparation of PCR reaction mixture, DNA preparations and PCR were carried out in facilities physically separate from each other (Tellevik et al., 2016). Sample analysis was done using Biosystems Cycler 7500 with a total reactional volume of 20 and 3 μl of DNA as described elsewhere for other protozoa with duplicates of positive and negative controls included in each run (Abu-Madi et al., 2015). PCR cycling conditions for *Cryptosporidum* spp. were 95°C for 10 min, followed by 50 cycles each cycle comprising 95°C for 15 s, 50°C for 30 s, and 72°C for 45 s each, and a final extension at 72°C for 2 min.

### Cryptosporidium geno/sub-typing

The RT-PCR positive products for *Cryptosporidium* spp. were subjected to restriction fragment length polymorphism (RFLP) analysis to identify the *Cryptosporidium* species (Coupe et al., 2005). Specimens that contained *Cryptosporidium parvum* or *Cryptosporidium hominis* were further subtyped by DNA sequencing of the PCR product of the gp60 gene (Sulaiman et al., 2005). Samples' sequences were aligned with reference sequences using the MAFFT program (EMBL) and BLAST searches were conducted with the edited sequences to determine the similarity of the samples with already published sequences. To determine the taxonomic positions, phylogenetic trees were constructed using the neighbor joining method by SplitsTree software, with robustness of groupings assessed using 1,000 bootstrap replicates of the data. The GP60 nucleotide sequences of the different subtypes were deposited in the GenBank database and the accession numbers are provided in the Results Section.

### Statistical analysis

Prevalence data are shown with 95% confidence limits (CL_95_), calculated as described by Rohlf and Sokal (1995) employing bespoke software. Prevalence was analyzed by maximum likelihood techniques based on log-linear analysis of contingency tables using the software package IBM SPSS Statistics (Version 23). Initially, full factorial models were fitted, incorporating as factors sex (SEX at 2 levels, males and females), age (AGE CLASS at 5 levels: Age class 1 (1 day–10 months), Age class 2 (11–20 months), Age class 3 (21–30 months), Age class 4 (31–60 months), and Age class 5 (>60 months); season [SEASON at 2 levels, winter (November–April) and summer (May–October)]; region of origin (REGION at 5 levels, as defined below). The children (*n* = 580) came from 37 countries which we allocated to five geographical regions for ease of initial analysis. These were Qatar (*n* = 168); Middle East (*n* = 92) comprising Bahrain (*n* = 3), Iraq (*n* = 6), Jordan (*n* = 23), Lebanon (*n* = 15), Oman (*n* = 2), Palestine (*n* = 3), Saudi Arabia (*n* = 6), and Syria (*n* = 17); and Yemen (*n* = 17); Africa (*n* = 119) comprising Djibouti (*n* = 1), Egypt (*n* = 88), Eritrea (*n* = 1), Libya (*n* = 1), Mauritania (*n* = 1), Morocco (*n* = 3), Nigeria (*n* = 1), South Africa (*n* = 1) Sudan (*n* = 10), and Tunisia (*n* = 12); Asia (*n* = 178) comprising Bangladesh (*n* = 6), China (*n* = 1), India (*n* = 87), Nepal (*n* = 3), Pakistan (*n* = 56), Philippines (*n* = 16), and Sri Lanka (*n* = 9); Other (*n* = 23) comprising children from other continents North America (*n* = 9), Great Britain (*n* = 3), Canada (*n* = 1), The Netherlands (*n* = 1), France (*n* = 3), Greece (*n* = 1), Italy (*n* = 1), Poland (*n* = 1), Spain (*n* = 2), and Venezuela (*n* = 1).

For each species of parasite, and for higher taxa, the presence/absence of parasites (INFECTION) was coded as a binary factor. The four explanatory factors listed above were fitted initially to all models that were evaluated. For each level of analysis in turn, beginning with the most complex model, involving all possible main effects and interactions, those combinations that did not contribute significantly to explaining variation in the data were eliminated in a stepwise fashion beginning with the highest-level interaction (the backward selection procedure). A minimum sufficient model was then obtained, for which the likelihood ratio of χ^2^ was not significant, indicating that the model was sufficient in explaining the data. The importance of each term (i.e., interactions involving INFECTION) in the final model was assessed by the probability that its exclusion would alter the model significantly and these values relating to interactions that included INFECTION are given in the text. Where relevant we also fitted in turn models with just one of the factors and INFECTION. A 2 × 2 χ^2^-test was implemented in some instances based on (Barnard et al., 2007).

Species richness (number of species harbored by each subject) was analyzed by generalized linear models (GLM) in SPSS 23, based on Poisson errors and we report the value of Wald χ^2^.

## Results

**Figure 1:**
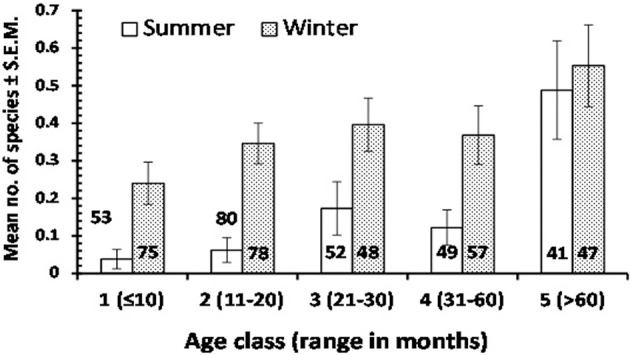
The mean no. of enteric protozoan species detected in children of varying age in summer and winter seasons. The sample size of each subset of data is given on the column or above the column to avoid obscuring the error bars. For statistical analysis see text.

Table 2 shows the distribution of subjects by region of origin and sex. Numbers in each age class are given in Figure 1. As can be seen, the largest category was children from Asia, and the smallest category was that referred to as “other” representing subjects from Europe and North and South America.

**Table 2:** Number of children in the study by country/region of origin and sex.

**Country/Region of origin**	**Sex**	**Number**	**Total by region**
Qatar	Male	93	
	Female	75	168
Middle East	Male	51	
	Female	41	92
Africa	Male	72	
	Female	47	119
Asia	Male	93	
	Female	85	178
Other^*^	Male	13	
	Female	10	23

* *This category included subjects from Europe, North and South America*.

### Overall prevalence

The prevalence of infection with each of the detected species is shown in Table 3. Overall, parasitic protozoa were detected in 22.9% of the children, and the most common species was *C. parvum*. Only two subjects harbored *Cryptosporidium meleagridis* and only one *E. histolytica*.

**Table 3:** Prevalence of protozoan parasites.

**Species/Taxon**	**%**	**95% CL**
Blastocystis hominis	4.7	3.43–6.26
*Giardia duodenalis*	1.7	1.05–2.83
*Dientamoeba fragilis*	4.0	2.85–5.49
*Entamoeba histolytica*	0.17	0.090–0.690
*Cryptosporidium* (species combined)	15.1	13.25–18.07
*C. parvum*	14.7	12.44–17.15
*C. hominis*	0.86	0.450–1.710
*C. meleagridis*	0.34	0.180–0.940
All species combined	22.9	20.25–25.85

### Effect of region of origin

The prevalence of combined protozoan infections varied significantly between regions (REGION × INFECTION, $\chi_{4}^{2}$ = 19.3, *P* = 0.001). Highest prevalence was recorded among Qatari children and the lowest among those from Asia (Table 4). An even stronger regional effect was found for *C. parvum* (REGION × INFECTION, $\chi_{4}^{2}$ = 40.5, *P* < 0.001), and again prevalence was highest among the Qataris. However, *B. hominis* showed similar prevalence among children from each of the five regions, varying only in the range 3.4–5.6%. For *D. fragilis* prevalence was relatively high among children from Europe and the Americas (Table 4), but there was no significant difference between the regions when tested in models that also took into account SEX, AGE, and SEASON.

**Table 4:** Prevalence of enteric protozoa in children, by region of their origin, age, sex, and season.

**Factor**			**Combined**		***C.parvum***		***B. hominis***		***D. fragilis***	
		***n***	**Mean**	**95%CL**	**Mean**	**95%CL**	**Mean**	**95%CL**	**Mean**	**95%CL**
**REGION**										
	Qatar	168	**33.9**	25.98–42.94	**26.8**	19.44–35.44	4.8	2.06–10.23	6.0	2.86–11.62
	Middle East	92	23.9	14.07–37.10	19.6	10.76–32.45	4.4	1.00–13.60	2.2	0.21–10.36
	Africa	119	17.7	12.51–24.16	10.1	6.27–15.52	3.4	1.43–7.25	1.7	0.48–5.04
	Asia	178	15.2	9.43–22.98	4.5	1.82–10.08	**5.6**	2.56–11.48	3.4	1.14–8.47
	Other	23	26.1	12.03–47.78	8.7	1.57–27.81	4.4	0.23–21.25	**13.0**	3.66–32.35
**SEX**										
	Males	322	19.6	15.57–24.30	13.7	10.29–17.86	3.1	1.66–5.63	**4.4**	2.57–7.15
	Females	258	**27.1**	22.97–31.72	**15.9**	12.59–19.79	**6.6**	4.5–9.47	3.5	2.05–5.76
**AGE**										
	Age Class 1	128	14.1	9.36–20.35	10.2	6.22–15.83	0.78	0.110–3.740	1.6	0.40–5.03
	Age Class 2	158	19.6	13.48–27.40	15.2	9.79–22.45	0.63	0.070–4.010	1.9	0.46–6.20
	Age Class 3	100	26.0	18.07–35.45	**18.0**	11.35–26.91	4.0	1.38–9.85	4.0	1.38–9.85
	Age Class 4	106	22.6	17.1–29.20	14.2	9.74–19.81	5.7	3.12–9.89	1.9	0.61–5.10
	Age Class 5	88	**38.6**	26.73–51.69	17.1	9.04–28.95	**17.1**	9.04–28.95	**13.6**	6.83–25.20
**SEASON**										
	Summer	275	12.0	9.05–15.68	4.0	2.40–6.47	**5.1**	3.28–7.80	**4.0**	2.40–6.47
	Winter	305	**32.8**	27.98–38.01	**24.3**	19.97–29.11	4.3	2.54–6.95	3.9	2.28–6.55

*The highest value in each data-subset is in bold*.

### Effect of host age

There was a highly significant difference in prevalence of combined protozoa across the age classes (AGE CLASS × INFECTION, $\chi_{4}^{2}$ = 18.8, *P* = 0.001), with the highest prevalence recorded among the oldest age class (61–174 month old; Table 4). The species with the greatest contribution to this age effect were *B. hominis* (Table 4; AGE CLASS × INFECTION, $\chi_{4}^{2}$ = 34.5, *P* < 0.001) and *D. fragilis* (AGE CLASS × INFECTION, $\chi_{4}^{2}$ = 19.7, *P* = 0.001). In contrast the prevalence of *C. parvum* varied only in the range from 10.0 to 18.0% (Table 4) among the 5 age classes.

### Effect of host sex

Prevalence of combined protozoa was significantly higher among female children (Table 4; SEX × INFECTION, $\chi_{1}^{2}$ = 4.6, *P* = 0.032), and this was to some extend attributable to the sex bias arising from *B. hominis* (SEX × INFECTION, $\chi_{1}^{2}$ = 3.9, *P* = 0.048) for which prevalence was twice as high among female compared with male children. There was no evidence of any sex bias for *C. parvum* or *D. fragilis* (Table 4).

### Effect of season

Prevalence of combined protozoan infection differed markedly and significantly between the seasons (Table 4; SEASON × INFECTION, $\chi_{1}^{2}$ = 36.9, *P* < 0.001), being 2.7 × higher in winter months compared with summer. A much greater seasonal effect was found for *C. parvum* (SEASON × INFECTION, $\chi_{1}^{2}$ = 5 3.0, *P* < 0.001) with prevalence in the winter months being 6 × higher than that in the summer (Table 4). However, no seasonal effect was evident for the prevalence of *B. hominis* or *D. fragilis* (Table 4).

### Other species

The other enteric protozoa recorded were too rare to analyze reliably. *G. duodenalis* was detected in ten children, five of whom were Asians, two African, two Qatari, and one from the Yemen in the Middle East. Six were females, seven of these infections were detected in the winter season and they were spread across all the age classes except the very youngest. Only one case of *E. histolytica* was recorded in a 71-month old (Age Class 5) Asian boy in the summer period. *C. hominis* was recorded in five subjects, three of whom were Asians, four were females, and three cases were in the winter. There were only two cases of *C. meleagridis*, both among age class 1 boys, both recorded in the winter, one from Africa and the other Qatari.

### Species richness

Table 5 summarizes mean values for species richness by each factor taken into consideration. Mean species richness was significantly affected by region of origin of the children (Wald $\chi_{4}^{2}$ = 11.1, *P* = 0.026), the highest value being derived from Qatari children and the lowest from African children. Additionally, mean species richness was affected by season (Wald $\chi_{1}^{2}$ = 22.1, *P* < 0.001) and by host age (Wald $\chi_{4}^{2}$ = 29.5, *P* < 0.001), but there was also a significant 2-way interaction between these latter two factors (SEASON × AGE CLASS on species richness, Wald $\chi_{4}^{2}$ = 12.7, *P* = 0.013), which is illustrated in Figure 1. This shows clearly that the age related increase in mean species richness was more rapid in the winter compared to in the summer months.

**Table 5:** Mean enteric protozoan species richness of children by nationality, age, sex, and season.

**Factor**		**Mean**	**SEM**
Region	Qatar	**0.40**	0.047
	Middle East	0.27	0.056
	Africa	0.18	0.035
	Asia	0.19	0.038
	Other	0.30	0.117
Age class	1	0.16	0.036
	2	0.20	0.033
	3	0.28	0.051
	4	0.26	0.048
	5	**0.52**	0.084
Season	Summer	0.15	0.029
	Winter	**0.36**	0.032
Sex	Male	0.24	0.03
	Female	**0.30**	0.032

*The highest value in each data-subset is in bold*.

### Polyparasitism

Among the 133 children infected with enteric protozoa, concurrent species infections were detected in only 16. The maximum number of species harbored by a single individual was 4 (one Asian boy, aged 71 months and tested in summer months, who was positive *for B. hominis, D. fragilis, E. histolytica*, and *C. hominis*). Two children harbored three species and 13 two species. The remaining 117 infected children were positive for only one species. Based on the prevalence of the seven individual species recorded (Table 3) in the entire study group, these numbers are much as predicted (Janovy et al., 1995) in the absence of interactions between species ($\chi_{6}^{2}$ = 3.06, *P* = 0.38; prediction = 438.9, 129.2, 11.5, 0.4, and 0, for uninfected, and those carrying 1, 2, 3, and 4 species, respectively).

**Figure 2:**
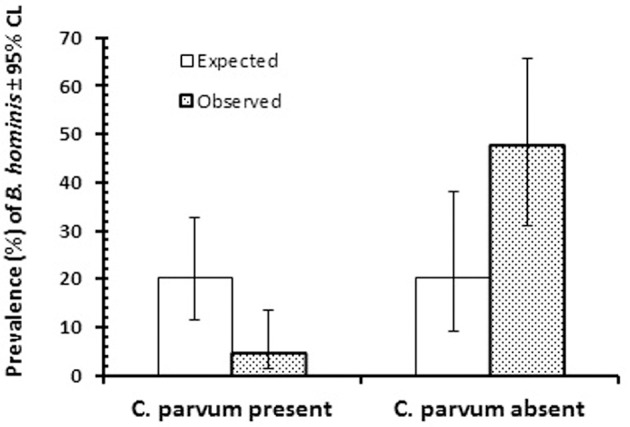
Prevalence of *B. hominis* among the 133 infected children that were infected with at least one of the enteric protozoa in the study. The figure shows prevalence of *B. hominis* among those in which *C. parvum* was also detected (*n* = 85) or was absent (*n* = 48). For statistical analysis see text.

However, when focusing on the two dominant species (*B. hominis* and *C. parvum*) among the 133 infected children (i.e., positive for at least one of the seven enteric protozoa detected in the study), some evidence for a negative association between these two species was evident, as illustrated in Figure 2. Based on a prevalence of 20.3% for *B. hominis* among the 133 children infected with at least one protozona species, prevalence among *C. parvum* infected children was markedly lower than expected, and accordingly higher among those without *C. parvum* infection (2 × 2 χ^2^-test, $\chi_{1}^{2}$ = 35.4, *P* < 0.0001). In a log-linear model, with AGE, SEX, REGION, and SEASON taken into account, there was also a significant association between prevalence of *B. hominis* and *C. parvum* in this subsample of children (INFECTION with *B. hominis* × INFECTION with *C. parvum*, $\chi_{1}^{2}$ = 27.3, *P* < 0.001), that was independent of any of other factors that were fitted to the model. However, when the whole data-set was analyzed (*n* = 580), prevalence of *B. hominis* among subjects with *C. parvum* [4.7% (1.28–13.59)] and those without [4.6% (2.41–8.46)] was almost identical.

Our data also revealed a significant positive relationship between *B. hominis* and *D. fragilis* (INFECTION with *B. hominis* × INFECTION with *D. fragilis*, $\chi_{1}^{2}$ = 22.9, *P* < 0.001) in the whole data-set (*n* = 580), and this retained significance when we controlled for REGION, AGE, SEX and SEASON ($\chi_{1}^{2}$ = 13.2, *P* < 0.001). Prevalence of *D. fragilis* was only 2.7% [1.83–4.00] among subjects without *B. hominis*, but more than 10-fold higher, 29.6 [14.77–50.00] among those with *B. hominis*. When analysis was confined to the 133 subjects that carried at least one protozoan species, prevalence of *D. fragilis* was also more than twice higher among *B. hominis* infected subjects [29.6% (14.77–50.00)] compared to those without *B. hominis* [14.2% (9.74–19.81)] but in this case the difference was not significant.

### *Cryptosporidium* geno/sub-typing

**Figure 3:**
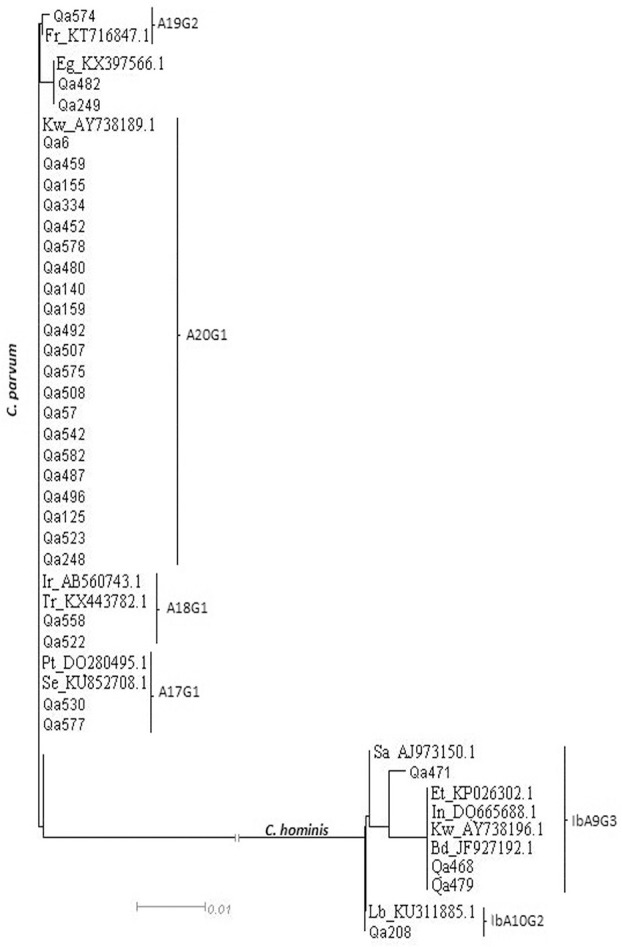
Phylogenetic analysis of some of the *C. hominis* and *C. parvum* subtypes using neighbor-joining analysis of the gylcoprotein 60 (gp60) gene. Reference sequences are preceded by indication of the country of origin.

A total of 90 samples, out of 580 that were positive by RT-PCR, were successfully genotyped. The 18S rRNA PCR-RFLP analysis revealed distinctive banding patterns. The majority of isolates [83/90 (92.22%)], were identified as *C. parvum*, making this the dominant species identified in the study population. Four samples (4.44%) were positive for *C. hominis*, one (1.11%) for *C. meleagridis*, one mixed (1.11%) *C. parvum* + *C. hominis*, and one mixed (1.11%) *C. parvum* + *C. meleagridis*. The GP60 gene was successfully sequenced in a total of 35 isolates, of which 31/35 (88.6%) were *C. parvum*, while 4/35 (11.4%) were *C. hominis* (Figure 3).

Alignments with reference sequences classified all *C. parvum* isolates into one family: IId. Further sub-classification led to 10 different subtypes of which the most frequently observed types were A20G1 (*n* = 22/ KY709197), A17G1 (*n* = 3; samples Qa342, Qa530, and Qa577/ Acc.num: KY709201), and A18G1 (*n* = 2; samples Qa522 and Qa 558/Acc.num: KY709200). A unique isolate was identified for each of the following sub-type: A19G2 (sample Qa574/Acc.num: KY709198), A18G2 (sample Qa58/Acc.num: KY709199), A16G1 (sample Qa249/Acc.num: KY709202), and A14G1 (sample Qa482/Acc.num: KY709203). The *C. hominis* subtype family identified among the four isolates was Ib. Within this subtype family, only two subtypes were identified with subtype IbA9G3 being the most common, present in three cases (samples Qa468, Qa471, and Qa479/Acc.num: KY709204, and KY709205) and subtype IbA10G2 identified in just one case (sample Qa208/Acc.num: KY709206).

## Discussion

While a range of different pathogenic organisms can cause pediatric diarrhea in Qatar, including rotaviruses and adenoviruses (Al-Thani et al., 2013) and foodborne bacteria such as, *Eschirichia coli* (Weam et al., 2016), in the current study we have shown that a significant percentage of cases of pediatric diarrhea (22.9%) is also associated with the presence of intestinal protozoan parasites. The most common among these was *C. parvum*, accounting for 14.7% of cases.

Cryptosporidiosis is globally a widespread disease with prevalence in humans reported to range between 1.1% in Iran (Tahvildar-Biderouni and Salehi, 2014) to more than 60% (El Shazly et al., 2007; Abdel-Hafeez et al., 2012). Prevalence of *B. hominis* in humans has been found to vary from 0.5 to 76% (Abu-Madi et al., 2015) and even as high as 100% among Senegalese children (El Safadi et al., 2014) and that of *D. fragilis* from 0.3 to 89% (Garcia, 2016). In our current work, we observed a prevalence of 4% for *D. fragilis* which is similar to prevalence values reported from Tunisia 5·5% (Ayadi and Bahri, 1999) and Australia 5% (Stark et al., 2016). Moreover, we also found evidence for a positive association between this species and *B. hominis* and this is consistent with reports on pediatric populations in Lebanon (Osman et al., 2016), and Portugal (Júlio et al., 2015).

Host intrinsic factors including age and sex have been assessed to identify potential factors that may influence the risk of infection with *B. hominis* in pediatric patients elsewhere (Nithyamathi et al., 2016) and in agreement with earlier studies, prevalence of infection with this species in our study was also found to increase significantly with host age. An age-effect was also detected for *D. fragilis*, however, there was no evidence in our data for an age effect in the case of *C. parvum*. The increase of infection prevalence with host age that we detected in the cases of *B. hominis* and *D. fragilis* may be related to changes in breast feeding practices with a reduction in exclusive breastfeeding as the children grow older. Although we did not find a significant increase in prevalence with host age in the case of *Cryptosporidium* spp., failure to breast feed is considered to be a risk factor for cryptosporidiosis (Aldeyarbi et al., 2016). Indeed mothers provide protective passive immunity against different parasites infection (Abdel-Hafeez et al., 2013; Pedersen et al., 2014), as reflected for example in the high levels of anti-*Cryptosporidium* IgA in maternal breast milk (Korpe et al., 2013). *G. duodenalis* infection has also been reported to be significantly less frequent among breastfed children (1.9%) compared with those not on breastfeeding (6.5%) in Egypt as well as among Rwandan children (Tellevik et al., 2016), but in our study the number of cases was too few to allow statistical analysis of any age effect in this species.

Prevalence of *B. hominis* was found to differ significantly between the sexes being twice as high among female children in contrast to observations elsewhere reporting higher prevalence among male subjects (Nithyamathi et al., 2016; Osman et al., 2016). The contrasting findings suggest that the distribution of intestinal protozoan infections is spatially heterogeneous with respect to host sex, and differences between studies are most likely linked with variation in local environmental conditions in the communities, and as a consequence with physiological properties intrinsic locally to hosts.

Intestinal protozoan infections are considered to be transmitted via the fecal-oral route, contaminated water/food (Anvari-Tafti et al., 2016), close contact with animals, poor personal hygiene and inadequate sanitation, and are facilitated by the environmentally resistant cyst stage of the parasites. Drinking contaminated water, especially surface water, is a known and significant risk factor for *B. hominis* infection (Abdulsalam et al., 2013). Cryptosporidiosis is also primarily spread by ingestion of contaminated water, and was ranked fifth among the 24 most important food-borne parasites (FAO, 2012). Among gastroenteritis outbreaks reported in England and Wales during the 1990s, *Cryptosporidium* spp. were identified as the probable cause in 14 outbreaks associated with public water supplies and swimming pools (Insulander et al., 2005; Suppes et al., 2016) as these species are resistant to chlorine at the levels usually used in water treatment (Maçin et al., 2016). The usage of surface water for irrigation can lead also to human infections through the consumption of contaminated fresh raw vegetables (FDA, 2015). Indeed, *Cryptosporidium* spp. have been detected on Cilantro leaves in Costa Rita and on lettuce in Norway (Sunnotel et al., 2006) and outbreaks of cryptosporidiosis have been observed to be associated with the consumption of fresh pre-cut salad leaves in England (McKerr et al., 2015) and fresh parsley in Sweden (Insulander et al., 2008). Green leafy vegetables such as, lettuce, rocket, and parsley have uneven surfaces and this provides a convenient substrate for the attachment of parasite eggs, cysts, and oocysts, either at the farms where they are grown or subsequently when washed with contaminated water (Said, 2012).

Our analysis has shown that prevalence of combined protozoan infections, and *C. parvum* in particular, was highest among the Qatari, despite Qatari nationals representing only 12% of the total population in the country (Qatar's Ministry of Development Planning and Statistics; Abu-Madi et al., 2016b). The most plausible routes for infection are via day-care centers (Clavel et al., 1996), swimming pools (Suppes et al., 2016), and food (as the consumption of salads is frequent in the country even in fast food restaurants). As children in Qatar have little contact with farm animals and the country relies on desalinated sea water that is piped to houses and used for drinking, these two potential routes for transmission are unlikely (Nazemalhosseini-Mojarad et al., 2012). A Portuguese study reported that the risk of acquiring this infection was 12 times greater for children who attended school, suggesting probable human-to-human transmission (Júlio et al., 2015). Foreign workers may also play a role in the transmission of intestinal parasites. In Saudi Arabia, pediatric parasitic infections (including *Cryptosporidium* spp.) have been suggested to be associated with the widespread custom of employing foreign maids who usually have direct contact with children. A high prevalence (46.5%) of intestinal parasites among female Asian housekeepers in that study, suggests that members of families with foreign housekeepers may be at some risk of parasitic infections (Ghenghesh et al., 2012).

As the etiological agents of diarrhoeal diseases occur naturally in both temperate and tropical areas, epidemics arising from the different agents may show specific seasonality. Meteorological conditions are known to have different effects on the transport, spread, reproduction, and persistence of a range of pathogens (Boithias et al., 2016). Concurring with our results, a recent pediatric study in Thailand, found a higher prevalence of intestinal parasites in the cool season compared with the hot season (Tellevik et al., 2016). In neighboring countries, such as, Kuwait, where high temperatures and a very dry atmosphere are typically experienced during the summer, and are followed by a short cool winter as observed in Qatar (Boughattas et al., 2017), prevalence of cryptosporidiosis also shows seasonality with highest prevalence during winter months (Iqbal et al., 2001). There is no regular rainy season in Kuwait or in Qatar, and thus the possibility of water-borne transmission attributable to natural sources of water is minimal. However, most studies relate seasonality of outbreaks of diarrhea positively with relative humidity (Jagai et al., 2009), whereas others report an opposite effect (Kiani et al., 2016) with correlation to water shortage and to greater reliance on less safe water sources (e.g., stored water) at certain times of the year. Interestingly, the period of high prevalence of infection in Kuwait has been reported to correspond to the “winter desert camping” season in the country when a large number of families spend an extended period of time in desert camps supplied with hundreds of overhead water tanks (Iqbal et al., 2011). A Spanish study reported marked seasonal variation in diarrhea, with peak prevalence in winter among 1-to 3-year-olds, which seemed to be related to attendance at child care centers, as the attendance was lowest in summer due to holidays (Clavel et al., 1996). Thus, this lack of consistency among seasonality studies and the absence of a straightforward relationship between ambient temperature, water quality and incidence of diarrhea highlight the complex relationship between hydro-meteorological and ecological factors and the transmission of waterborne diseases.

The distribution of *Cryptosporidium* spp. in humans is known to vary with geographic region and also with the socioeconomic conditions of the study population. Our findings are consistent with previous reports from other Middle Eastern countries (Nazemalhosseini-Mojarad et al., 2012). In Saudi Arabia, *C. parvum* was identified in 43 of 53 examined samples (Alyousefi et al., 2013) and in Kuwait *C. parvum* was the predominant causative agent of cryptosporidiosis in children (Sulaiman et al., 2005; Iqbal et al., 2011). Gp60 subtyping has shown that not all *C. parvum* infections in humans are of zoonotic transmission (Xiao, 2010). Within each subtype family, one subtype is frequently seen to dominate in specific communities/countries. For example, in industrialized countries most of *C. hominis* infections are caused by the Ib subtype family (Waldron et al., 2009), which appears to be the most virulent as reflected by the numerous outbreaks with which it has been linked. Worldwide there are two common subtypes within the subtype family Ib: IbA9G3 and IbA10G2. IbA9G3 is commonly seen in Malawi, Kenya, and India, whereas IbA10G2 is commonly seen in South Africa, Botswana, Jamaica, and Peru (Xiao, 2010). Using GenBank BLASTN, a similar IbA9G3 subtype to that identified in our isolates has been observed among children in North India (Gatei et al., 2007) and Bangladesh (Hira et al., 2011). The IbA10G2 subtype was reported to be involved in waterborne outbreaks especially in Sweden (Gherasim et al., 2012) and almost half of the *C. hominis* outbreaks in USA (Cacciò and Chalmers, 2016). It has been also associated with swimming pool outbreaks in Australia (Ng-Hublin et al., 2015).

Sequence analysis of the GP60 locus in the present study has identified only *C. parvum* subtype family IId. The source of the IId subtype in humans is not yet clear. It is the dominant *C. parvum* subtype family in humans in Middle Eastern countries such as, Iran, Syria (Kassouha et al., 2016), and Saudi Arabia (Nazemalhosseini-Mojarad et al., 2012). The most frequent subtype observed in our samples was the IIdA20G1 as identified predominantly in Egypt, Jordan (Helmy et al., 2013; Hijjawi et al., 2016), and among Kuwaiti children (Sulaiman et al., 2005; Iqbal et al., 2011). Recurrently identified in our population, subtype IIdA17G1, was reported to be the cause of an outbreak in Finland in 2012, with more than 250 individuals involved and it was associated with consumption of fries and salads (Cacciò and Chalmers, 2016), whereas subtype IIdA18G1 has been widely identified in Europe (e.g., Czech Republic, Spain) and in the Middle East (e.g., Turkey, Iran, Kuwait, etc.). Less frequently observed, subtype IIdA19G2 has been detected in France as a foodborne etiological agent (GenBank number KT716847.1), IIdA14G1 in Spain (Plutzer and Karanis, 2009), and IIdA16G1 in Egypt and Tunisia (Rahmouni et al., 2014). *C. meleagridis*, mainly isolated from poultry and birds, has been frequently observed in Africa (Aldeyarbi et al., 2016) as well as within mixed cryptosporidium infections (Ng-Hublin et al., 2013). Although, as stated earlier, Qatari children have limited contact with wildlife, such animal subtypes may infect them indirectly through housemaids who themselves may have been exposed to animals back home and/or through their presence in the food supply via remnants of fertilizers of animal origin on salad crops.

## Conclusion

In our previous work, we suggested that transmission of some protozoa does occur in Qatar and our current results support this hypothesis based on the high rate of infection among Qatari subjects. Cryptosporidiosis has been shown to be the most frequent pediatric diarrheic infection. Food borne infective stages and a human-to-human role appear to constitute the most probable routes of transmission. In order to better understand this parasite's circulation among the population of Qatar, further studies targeting housemaids and food handlers are required.

## Author contributions

SB carried out the experiments and drafted the manuscript; JB carried out the statistical analysis and participated in the preparation and refinement of the manuscript; KA collected the samples and their background information; AS contributed to the molecular detection; WA contributed in sequencing investigation; AA contributed to the interpretation of the results and participated in refinement of the manuscript; MA conceived the study, collected the data and revised the manuscript critically for important intellectual content. All authors read and approved the final manuscript. The contents of this report are solely the responsibility of the authors and do not necessarily represent the official views of Qatar University and QRNF.

### Conflict of interest statement

The authors declare that the research was conducted in the absence of any commercial or financial relationships that could be construed as a potential conflict of interest.
